# RAD sequencing resolved phylogenetic relationships in European shrub willows (*Salix* L. subg. *Chamaetia* and subg. *Vetrix*) and revealed multiple evolution of dwarf shrubs

**DOI:** 10.1002/ece3.4360

**Published:** 2018-07-22

**Authors:** Natascha Dorothea Wagner, Susanne Gramlich, Elvira Hörandl

**Affiliations:** ^1^ Department of Systematics, Biodiversity and Evolution of Plants (with Herbarium) University of Goettingen Göttingen Germany

**Keywords:** character evolution, phylogenetics, RAD sequencing, *Salix*

## Abstract

The large and diverse genus *Salix* L. is of particular interest for decades of biological research. However, despite the morphological plasticity, the reconstruction of phylogenetic relationships was so far hampered by the lack of informative molecular markers. Infrageneric classification based on morphology separates dwarf shrubs (subg. *Chamaetia*) and taller shrubs (subg. *Vetrix*), while previous phylogenetic studies placed species of these two subgenera just in one largely unresolved clade. Here we want to test the utility of genomic RAD sequencing markers for resolving relationships at different levels of divergence in *Salix*. Based on a sampling of 15 European species representing 13 sections of the two subgenera, we used five different RAD sequencing datasets generated by ipyrad to conduct phylogenetic analyses. Additionally we reconstructed the evolution of growth form and analyzed the genetic composition of the whole clade. The results showed fully resolved trees in both ML and BI analysis with high statistical support. The two subgenera *Chamaetia* and *Vetrix* were recognized as nonmonophyletic, which suggests that they should be merged. Within the *Vetrix/Chamaetia* clade, a division into three major subclades could be observed. All species were confirmed to be monophyletic. Based on our data, arctic‐alpine dwarf shrubs evolved four times independently. The structure analysis showed five mainly uniform genetic clusters which are congruent in sister relationships observed in the phylogenies. Our study confirmed RAD sequencing as a useful genomic tool for the reconstruction of relationships on different taxonomic levels in the genus *Salix*.

## 1 INTRODUCTION

**Figure 1:**
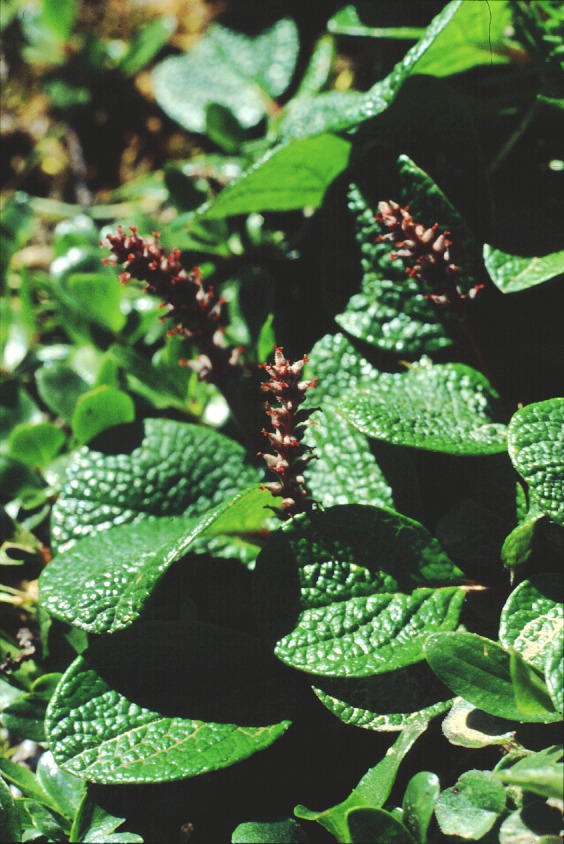
Example of the dwarf shrub growth form in willows: *Salix reticulata* (subg. *Chamaetia*) is a circumpolar arctic‐alpine species and only few centimeters high. In the picture, it shows flowering female catkins

The genus *Salix* L. (Salicaceae) comprises about 400–450 species of trees and shrubs mainly occurring in the Northern Hemisphere with a distribution center in China (Argus, 1997; Skvortsov, 1999). Willows are important elements of various kinds of natural wetlands, riparian vegetation, and arctic‐alpine tundras and are involved in many biotic interactions (e.g., Hörandl, Florineth, & Hadacek, 2012; Pasteels & Rowell‐Rahier, 1992; Sommerville, 1992). Many species of *Salix* are of economic importance for usage in soil engineering, landscape gardening, as ornamental plants or for biomass production (Hörandl et al., 2012; Newsholme, 1992; Schiechtl, 1992). Despite the ecological and economic importance of the genus, the taxonomy and systematics in *Salix* have proven to be extremely difficult because of dioecious reproduction, simple, reduced flowers, common natural formation of hybrids, formation of polyploids, and large intraspecific phenotypic variation (Cronk, Ruzzier, Belyaeva, & Percy, 2015; Hörandl et al., 2012; Skvortsov, 1999). In particular, the latter point led to the description of many species and the overall taxonomy of *Salix* is still far from resolved (Dickmann & Kuzovkina, 2014). Nevertheless, based on morphological characters, the genus is divided into three (or five) subgenera: *Salix* subg. *Salix* s.l. (including subgenera *Salix* L., *Longifoliae* (andersson) argus *, Protitae* kimura, or excluding the latter two), subg. *Chamaetia* (dumort). nasarov in kom., and subgen. *Vetrix* dumort. (Argus, 2010; Lauron‐Moreau, Pitre, Argus, Labrecque, & Brouillet, 2015; Skvortsov, 1999; Wu et al., 2015). In Eurasia, subgenera *Chamaetia* and *Vetrix* comprise 19 sections and 51 species (based on Skvortsov, 1999). Species of subgenus *Chamaetia* are adapted to cold, hostile environments of the arctic and alpine zone including small and dwarf shrubs. These species show a decumbent or creeping growth, forming mats directly on the ground (*S*. *reticulata* (Figure 1), *S. herbacea*), dense cushions (*S. serpillifolia*), or small decumbent shrubs like *S. breviserrata* with flowering shoots ascending up to 30 (max. 50) cm. This alpine dwarfism is adaptive for arctic‐alpine woody plants (Körner, 2003) and also remains stable under cultivation in the lowlands (Newsholme, 1992; Schiechtl, 1992). Based on their morphological and ecological similarity, the five sections of these highly specialized dwarf willows are summarized in subgenus *Chamaetia* (Skvortsov, 1999). Subg. *Vetrix* comprises medium sized to tall shrubs and trees. However, the morphological characters used to distinguish the two subgenera are not exclusive but show transitions, and although widely accepted, the morphology‐based separation into two distinct subgenera was and still is subject to discussion (Argus, 1997; Skvortsov, 1999).

Despite the huge interest in *Salix*, the morphological classification is lacking any molecular support. In general, the phylogenetic relationships among willow species are still poorly understood, for molecular studies mainly used traditional Sanger sequencing markers like ITS or plastid regions that lack phylogenetic signal (Azuma, Yokohama, & Ohashi, 2000; Barcaccia, Meneghetti, Albertini, Triest, & Lucchin, 2003; Chen, Sun, Wen, & Yang, 2010; Lauron‐Moreau et al., 2015; Leskinen & Alström‐Rapaport, 1999; Percy et al., 2014; Savage & Cavender‐Bares, 2012; Wu et al., 2015). Although these studies were able to confirm the monophyly of the genus and to separate a small, basal clade of subtropical to temperate trees (subg. *Salix* s.l.), they failed to resolve the relationships of the shrub species classified as subgenera *Chamaetia* and *Vetrix* (Azuma et al., 2000; Chen et al., 2010; Leskinen & Alström‐Rapaport, 1999; Savage & Cavender‐Bares, 2012). Percy et al. (2014) analyzed seven plastid markers and were not able to delimit taxonomic species based on the plastid phylogenies. They found wide spread sharing of few haplotypes among species and sections, for example, all accessions of subg. *Chamaetia* belong to one haplotype, while sections and species of subg. *Vetrix* belong to more than one plastid haplotype. The authors explained the low species‐specific identity by horizontal gene transfer, frequent chloroplast capture, and trans‐specific selective sweeps. A more recent study of Wu et al. (2015) focused on relationships of *Salix* subg. *Salix* s.l. using a combination of four plastid and two nuclear markers. Accessions of subgenera *Chamaetia* and *Vetrix* form a clearly monophyletic clade, but with no or low interspecific resolution. The authors detected conflicting phylogenetic signals and regarded ancient hybridization and introgression as the main reasons. A study of Lauron‐Moreau et al. (2015) also revealed reticulate evolution among the clades of subgenera *Salix, Protitea,* and *Longifolia*. However, hybridization is an extensively reported phenomenon in *Salix* and occurs even between distantly related species of different subgenera (Argus, 2010; Hörandl et al., 2012; Skvortsov, 1999). The *Vetrix‐Chamaetia* clade started to diversify in the late Oligocene (23.7 mya; Wu et al., 2015), but more comprehensive sequencing data suggest that its major radiation may coincide with Quaternary radiations (Lauron‐Moreau et al., 2015). Hence, we regard ancient reticulate evolution and conflicting phylogenetic signals a plausible hypothesis for the observed unresolved tree topologies.

A study focusing on European willows, especially including a comprehensive sampling of the *Chamaetia/Vetrix* clade, is missing. The lack of any solid phylogenetic framework for this big clade hampered the understanding of evolution and delimitation of taxa. However, a well‐resolved molecular phylogeny would create new opportunities for overcoming the difficulties of working with this taxonomic difficult group and to shed some light on the evolution of European shrub willows.

Next‐generation sequencing offers powerful tools for resolving relationships within and among closely related species that lack resolution with traditional markers (Davey et al., 2011; Eaton & Ree, 2013; Emerson et al., 2010; Etter, Preston, Bassham, Cresko, & Johnson, 2011; Hohenlohe et al., 2010; Hörandl & Appelhans, 2015). One of these tools is *R*estriction‐site *A*ssociated *D*NA (RAD) sequencing (Baird et al., 2008), which is a frequently used reduced representation method to generate thousands of informative markers for many samples at the same time for comparatively low costs (reviewed in Andrews, Good, Miller, Luikart, & Hohenlohe, 2016). This method combines enzymatic fragmentation of genomic DNA with high‐throughput sequencing methods. Established mainly for population genetics (Baird et al., 2008; Baxter et al., 2011), it is nowadays used for species delimitation in closely related groups (e.g., Herrera & Shank, 2016; Pante et al., 2015; Wang et al., 2013) as well as genus‐wide analyses (e.g., Eaton & Ree, 2013; Vargas, Ortiz, & Simpson, 2017). Also deep‐scale phylogenies are possible using RAD sequencing data (Eaton, Springs, Park, & Donoghue, 2017). Many recent studies have used RAD sequencing data to resolve phylogenetic relationships in groups where Sanger sequence‐based data have failed due to insufficient variation (e.g., Eaton & Ree, 2013; Herrera & Shank, 2016; Jones, Fan, Franchini, Schartl, & Meyer, 2013; Wagner et al., 2013). Therefore, RAD sequencing seems a promising tool to resolve relationships within complicated genera like willows (*Salix*).

In this study, we attempt the advantages of RAD sequencing to analyze a set of European shrub willows (*Chamaetia/Vetrix*) covering 13 sections to overcome the lack of information on relationships within this interesting group. As no study was published so far that used RAD sequencing for willow phylogenetics, we aim to test the utility of this method for uncovering relationships on three different levels of divergence: (a) between and within the two subgenera, (b) between and within sections, and (c) for the delimitation of species. Based on the resulting phylogeny, we want to (d) reconstruct character evolution of growth habit as a traditional diagnostic character for taxonomy.

## 2 MATERIAL AND METHODS

### 2.1 Sampling

For this study, we sampled 13 diploid species and one triploid species (*S. bicolor*) representing 13 sections of the two subgenera *Chamaetia* and *Vetrix*. *Salix triandra* (subg. *Salix*, Section *Amygdalinae* koch) was included to serve as outgroup (as closest relative following the results of Wu et al., 2015) resulting in a total of 15 species. The samples were collected in Central and Northern Europe and determined after Skvortsov (1999) and Hörandl et al. (2012). Leaves were dried in silica gel, and herbarium voucher specimens were deposited in the herbarium of the University of Goettingen (GOET). To reconstruct a basic phylogenetic framework without confounding effects of polyploidy, we reduced our sampling to diploid species only, except *S. bicolor*, which is reported to be triploid (Dobeš & Vitek, 2000). In almost all cases, four accessions per species were included in the analyses given a total of 58 samples. Detailed information about the sampling is summarized in Supporting Information Table S1. A phylogenetic study based on a comprehensive sampling, including also polyploids, will be presented elsewhere.

### 2.2 Molecular treatment and analyses

The DNA of all 58 samples including four *S. triandra* accessions as outgroup was extracted using the Qiagen DNeasy Plant Mini Kit following the manufacturer′s instructions (Valencia, CA). After quality check the DNA was sent to Floragenex, Inc. (Portland, Ore., USA) where the RAD sequencing library preparation was conducted after the protocol described in Baird et al. (2008). The methylation‐sensitive restriction enzyme *Pst*I was used for digestion. After size selection of 300 bp–500 bp with a Pippin Prep (Sage Science, Beverly, Massachusetts, USA), the libraries were barcoded by individual and multiplexed on an Illumina HiSeq 2500 (Illumina Inc., San Diego, CA, USA). Quality of the resulting sequences was checked using fastQC v.0.10.1 (Andrews, 2010).

Sequence reads were de‐multiplexed, and the fastq files of each sample were used to run ipyrad v.0.6.15 (Eaton & Overcast, 2016). The adapter trimming option included in ipyrad was used to make sure that all adapters are removed. Reads were clustered within each individual by similarity of 85% using the implemented vclust function in vsearch (Edgar, 2010). Clusters with less than six reads were excluded in order to ensure accurate base calls. The consensus sequences of each individual were clustered across samples by sequence similarity of 85%. The resulting clusters represent putative RAD loci shared across samples. To test the effects of different parameter settings in the pipeline, datasets resulting from five different thresholds for the minimum number of samples per locus (mc) were performed. In particular, we conducted analyses with a “full” dataset setting the minimum number of samples per locus to four because we included four individuals per species (mc4), and a “reduced” dataset of loci shared by all individuals (mc58). Additionally, we used mc20, mc35, and mc50 as intermediate thresholds. The number of maximal shared heterozygotic sites and indels set to five was used to receive the total number of loci for the subsequent analyses.

We inferred phylogenetic relationships on concatenated alignments using maximum likelihood (ML) using the GTR+ Γ model of nucleotide substitution implemented in RaxML v.8.2.4 (Stamatakis, 2014) and performed for each analysis a rapid bootstrapping analysis with 100 replicates using the –f a option, which searches for the best‐scoring tree (Stamatakis, Hoover, & Rougemont, 2008). Additional phylogenetic analyses were performed based on Bayesian Inference (BI) using mrbayes v.3.2.6 (Ronquist & Huelsenbeck, 2003). Every 1,000 step was sampled on an analysis of 1,000,000 generations with four MCMC chains (heating parameter = 0.05) in two independent runs using the GTR+ Γ substitution model. We discarded 25% as burn‐in and used the remaining samples to compute a majority consensus tree. The resulting trees of all analyses were obtained in figtree v1.4.3 (Rambaut, 2014).

In order to test for an influence of reticulate evolution on the genetic composition of the included species, we performed an analysis in structure v. 2.3.4 (Pritchard, Stephens, & Donnelly, 2000) on the complete dataset. Additionally, because of the high divergence, we performed an analysis without the outgroup *S. triandra*. The structure output format of unlinked SNPs (.ustr) of the ipyrad pipeline for the mc20 dataset was used. We chose a burn‐in of 5,000 and a MCMC of 50,000 replicates, with three replicates of each value of *K* (*K*=number of genotypic groups). The range of *K* was set from 2 to 13. The optimal *K* value was estimated using the delta *K* value in structure harvester (Earl & vonHoldt, 2012).

To analyze the evolution of growth form within the European shrub willows, an ancestral character state approach was performed using mesquite 3.31 (Maddison & Maddison, 2017). For tracing the evolutionary history, we did parsimony reconstructions. We used a character matrix containing three character states, that is, “dwarf shrub” (<50 cm height, decumbent, mat‐forming), “medium‐sized shrub” (50–150 cm, erect or ascending), and “large shrub/tree” (>150 cm, erect), respectively (Figure 4). Categorization of species followed Skvortsov (1999) and Hörandl et al. (2012). The variability of the growth within species falls well within these broadly defined three character states even in different areas, as reported from Elven and Karlsson (2000) for Northern Europe and Rechinger (1957) for the Alps. Dwarf growth of the high alpine/arctic species remains also stable in cultivation in lowlands, where some of the species are popular rock garden plants (Newsholme, 1992) or are used for soil engineering and landscape gardening (Schiechtl, 1992). The analyses were based on the ML tree of the mc20 dataset.

## 3 RESULTS

### 3.1 RAD sequencing

An average of 8,549,092 (standard deviation ± 2,535,025) Illumina reads per sample was generated. After quality filtering, an average of 8,375,879 filtered reads was used for the ipyrad pipeline. It revealed a total of 293,444 prefiltered RAD loci. After filtering steps, the number of retained loci varied between 2,051 (mc58) and 68,499 (mc4). 2,051 loci were shared by all 58 taxa included (incl. outgroup, Table 1). The average read depth per locus was 37.63 reads. The number of parsimony informative sites (PIS) differed between 5,098 (mc58) and 63,497 (mc4).

**Table 1:** IPYRAD statistics for five different datasets with different minimum number (mc) of taxa per locus (mc4, mc20, mc35, mc50, mc58) containing 58 samples representing 15 *Salix* species. The total number of prefiltered loci is 293,444

Datasets	mc4	mc20	mc35	mc50	mc58
Total filtered loci	68,499	6,035	3,990	3,406	2,051
Total variable sites (SNPs)	124,611	22,054	15,070	12,855	7,564
Parsimony informative sites (PIS)	63,497	14,171	9,966	8,591	5,098
Supermatrix (bp)	5,533,110	491,120	324,001	276,483	166,449
Amount of missing data (%)	83,73	23,81	6,35	2,77	0

*Note*

bp: basepairs.

### 3.2 Phylogenetic analyses

**Figure 2:**
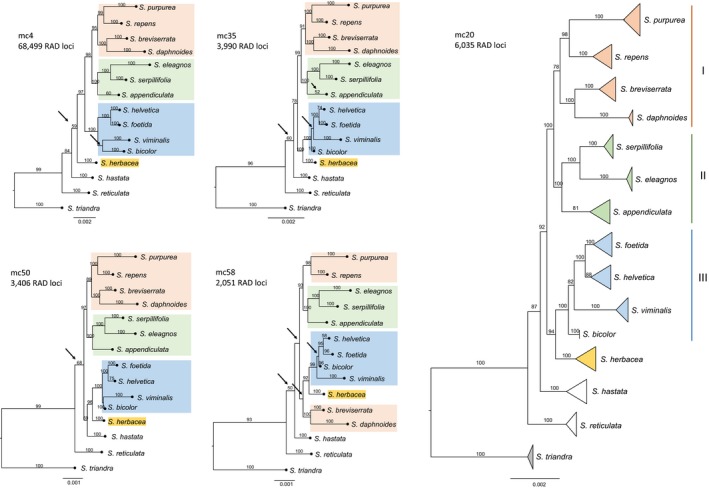
Comparison of simplified ML tree topologies based on different minimum number of samples per locus (mc4, mc20, mc35, mc50, and mc58). Clades I, II, III indicated by colors. The different positions of *Salix herbacea* highlighted in yellow. Bootstrap values (>50) above branches, low/no support (<75) indicated by arrows. Trees were rooted with *Salix triandra* as outgroup

**Figure 3:**
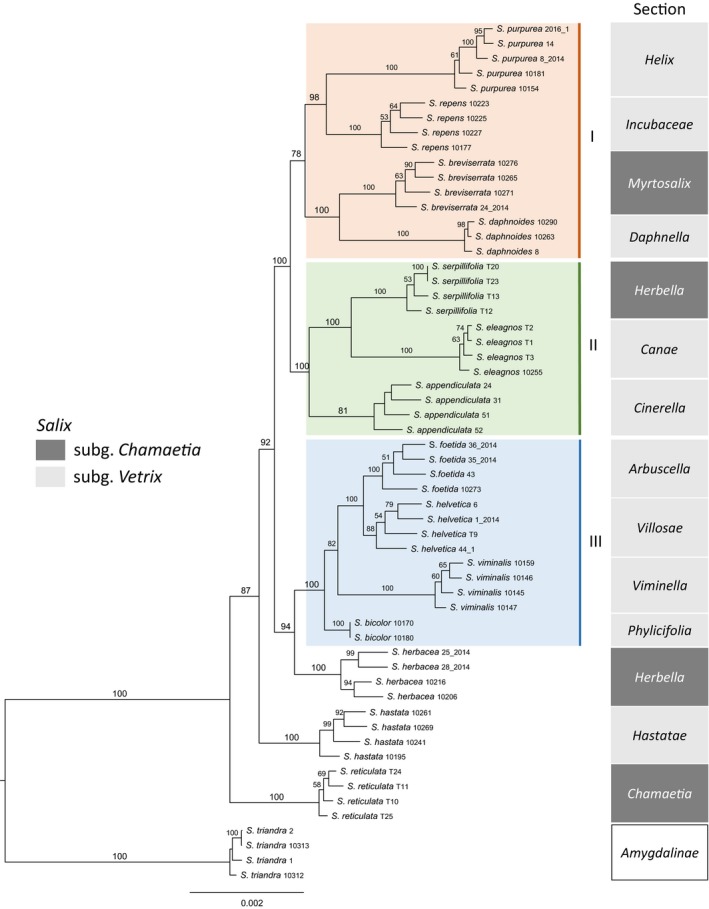
Detailed maximum‐likelihood phylogeny based on the mc20 dataset. *Salix triandra* was used as outgroup. The three main clades I, II, III are indicated by colored boxes. Sections of subgenus *Chamaetia* are marked in dark grey, of subgenus *Vetrix* in light gray. Bootstrap values (>50) above branches

The results of all different datasets (mc4, mc20, mc35, mc50, mc58) yielded substantial resolution of the European shrub willows and are presented in Figure 2. The resulting trees of the ML analyses for the mc20, mc35, and mc50 are almost identical in topology and differ only in statistical support (Figure 2). The ML phylogenies based on the “full” (mc4) and “reduced” (mc58) dataset show slightly different topologies (Figure 2). The trees inferred from BI for the m20, m35, m50, and m58 datasets are identical in topology with the RAxML analyses of the same datasets (Supporting Information Figure S1). We will continue describing the well‐resolved ML tree based on the mc20 dataset (Figure 3), which has the highest Bootstrap (BS), and in the equivalent BI analysis the highest posterior probability (PP) support values. All species are clearly monophyletic and well supported (BS 100, PP 1). All members of the *Chamaetia/Vetrix* clade form a well‐supported monophyletic group (BS 100, PP 1). *Salix reticulata* appears as an early diverging taxon in sister position to all remaining *Salix* species, followed by a split of *S. hastata*. The remaining accessions form three monophyletic groups. Clade III (BS 100, PP 1) contains *S. helvetica*,* S. foetida*,* S. viminalis,* and *S. bicolor*. This clade is situated together with *S. herbacea* in sister position (BS 94, PP 1) to the remaining two clades, I and II. They contain *S. purpurea*,* S. repens*,* S. breviserrata,* and *S. daphnoides* (clade I, BS 78, PP 1) and *S. serpillifolia*,* S. eleagnos,* and *S. appendiculata* (clade II, BS 100, PP 1), respectively. In the mc4 dataset based on 68,499 loci, *S. herbacea* is in sister position to the three main clades. In the mc58 dataset, the clade I is polyphyletic: *S. purpurea* and *S. repens* are sister to clade II, whereas the other two species, *S. breviserrata* and *S. daphnoides,* are in an unsupported sister position to clade III plus *S. herbacea*. The backbone of this phylogeny reaches low to no support. Despite the good statistical support of the clades and species, they do not reflect the traditional taxonomic classification. The subgenera and sections are mapped onto the phylogeny in Figure 3. Neither subgenus *Chamaetia* nor *Vetrix* is monophyletic on its own. Furthermore, the accessions of two species of the section *Herbella*, that is, *S. herbacea* and *S. serpillifolia,* occur in two different clades.

### 3.3 Character evolution

**Figure 4:**
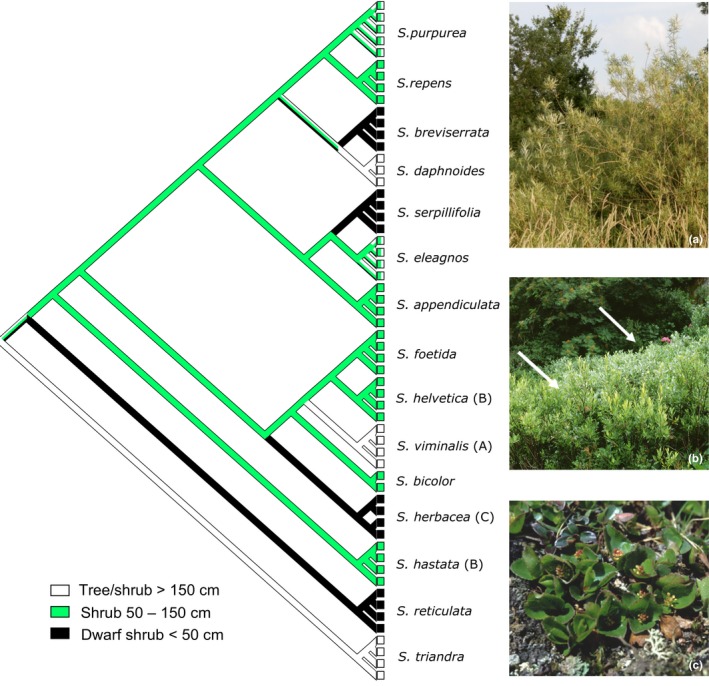
Results of character evolution analysis of growth habit based on the ML results of the mc20 dataset. The character coding includes large shrubs/trees (>150 cm, indicated in white), medium‐sized shrubs (50–150 cm, indicated in green), and dwarf shrubs (<50 cm, indicated in black). The analysis in mesquite was performed using the Maximum Parsimony option. On the right hand side, examples of the three different growth forms are given. Lowland tall shrubs and trees >150 cm (A, *Salix viminalis*), subalpine shrubs between 50–150 cm (B, *S. hastata* (left arrow), and *S. helvetica* (right arrow)) and arctic‐alpine dwarf shrubs <50 cm (C, *S. herbacea*)

Based on the ML of the mc20 dataset, we conducted a character evolution analysis in mesquite. The results show an at least fourfold independent evolution of dwarf shrubs (Figure 4). This growth habit occurs within each of the three major clades: *S. breviserrata* in clade I, *S. serpillifolia* in clade II, and *S. herbacea* as sister to clade III. In each case, the dwarf shrubs are in sister position to a medium‐sized shrub or even tree‐forming species. Interestingly, the alpine dwarf shrub *S. reticulata* is sister to all other included European taxa in the ML and BI phylogeny.

### 3.4 Genetic structure analysis

**Figure 5:**
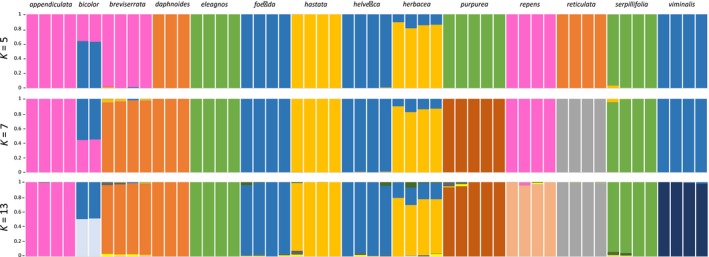
Results of the structure analysis of the mc20 dataset of unlinked SNPs for the most likely *K* value (*K* = 5) as well as *K* = 7 (second likeliest *K*) and *K* = 13 (because 13 sections were included). Similar genetic clusters are indicated by the same color. Each bar represents one individual, *Salix* species names given above bar blots. The four outgroup taxa of *S. triandra* were excluded from the analysis. The included accessions show a more or less uniform pattern according their species delimitation except *S. bicolor*, which is triploid

The structure output file of unlinked SNPs (.ustr) from ipyrad was used directly for the genetic structure analyses. Following the results of structure harvester, the bar plot for the most likely population size of *K* = 5 is shown in Figure 5. Additionally, *K* = 7 (second highest probability) and *K* = 13 (for 13 sections were included) are shown for comparison. The analysis shows for all diploid species a uniform genetic structure. Only in *S. herbacea* a little admixture is present (Figure 5). According to *K* = 5, five genetic clusters can be observed: *S. appendiculata*,* S. breviserrata,* and *S. repens* share the same structure (pink). The same is true for *S. daphnoides* and *S. reticulata* (indicated in orange) and *S. eleagnos*,* S. purpurea,* and *S. serpillifolia* (indicated in green). Finally, *S. foetida, S. helvetica,* and *S. viminalis* form a genetic cluster (identical with clade III in the phylogenies, blue) as well as *S. hastata* and *S. herbacea* (yellow). The triploid species *S. bicolor* shows genetic admixture with about two‐third contribution of the clade III (blue) and one‐third of the “pink group” for *K* = 5. These clusters do not exactly reflect the clades of the ML and BI phylogenies, but are congruent in sister relationships (e.g., *S. foetida* and *S. helvetica* or *S. eleagnos* and *S. serpillifolia*). Looking at the results of higher K‐values, the observed clades become more evident. For instance, with a setting of the number of populations to the second likely value of *K* = 7 and to *K* = 13 (Figure 5), *S. viminalis*,* S. foetida,* and *S. helvetica* share the same genetic structure, similar to *S. bicolor* (clade III). The same is true for *S. breviserrata* and *S. daphnoides* (part of clade I), and for *S. serpillifolia* and *S. eleagnos* (clade II).

## 4 DISCUSSION

### 4.1 Utility of RAD sequencing for willow phylogenies

In this study, we used RAD sequencing to test its utility for resolving phylogenetic relationships in diploid members of the European shrub willows. The resulting trees were all fully resolved, indicating that this tool is informative for both deep and shallow levels of divergence. Traditional sequencing markers failed to resolve relationships in the genus *Salix* subg. *Chamaetia/Vetrix* (Chen et al., 2010; Lauron‐Moreau et al., 2015; Savage & Cavender‐Bares, 2012; Wu et al., 2015). One strategy to overcome this problem of a lack of resolution is to collect as many independent genomic markers as possible. RAD sequencing generates thousands of informative characters distributed over the whole genome that provide enough information to resolve interspecific relationships. Thus, many studies have used RAD sequencing methods to resolve phylogenetic relationships in groups where Sanger sequence‐based data have failed due to insufficient variation, gene tree discordance, or both (e.g., Eaton & Ree, 2013; Ebel et al., 2015; Escudero, Eaton, Hahn, & Hipp, 2014; Jones et al., 2013; Herrera & Shank, 2016; Hipp et al., 2014; Mort et al., 2015; Vargas et al., 2017; Wagner et al., 2013; Wang et al., 2013). When investigating more divergent taxa with restriction digestion methods, the high amount of loci drop out and subsequently missing data may create difficulties (e.g., Cariou, Duret, & Charlat, 2013; Lemmon & Lemmon, 2013; Leaché, Banbury, Felsenstein, Nieto‐Montes de Oca, & Stamatakis, 2015; Ree & Hipp, 2015; Andrews et al., 2016; Huang & Knowles, 2016). However, Eaton et al. (2017) showed that missing data in RAD sequencing has different reasons and only minor effects on phylogenetic reconstruction. The authors suggest that sufficient sequencing depth increases the phylogenetic utility of RAD sequencing datasets. Here we included at least four accession per sample and observed an average of more than 8 Mio high‐quality reads to guarantee sufficient sequencing coverage and phylogenetic information.

The total number of loci (and SNPs) necessary to resolve a phylogenetic tree is dependent on the evolutionary distance of the included taxa and the variability of loci. In our analyses, we compared five different thresholds to check for statistical support and robustness of the topology on different levels of divergence. The mc20, mc38, and mc50 datasets show an almost identical topology for both algorithms (Figure 2, Supporting Information Figure S1) although mc50 was based on about half of the number of loci than the mc20 dataset (Table 1). Only the statistical support values differ, whereby highest bootstrap values and posterior probabilities, respectively, on all levels were observed in the mc20 dataset with 6,025 loci. The topology of the phylogenetic tree based on 2,051 loci shared by all individuals (mc58) is different: clade I is not monophyletic but split into two minor clades: *S. purpurea* and *S. repens* group as sister to clade II and *S. breviserrata* and *S. daphnoides* as sister to clade III + *S. herbacea*, respectively. The BS support of this tree is generally very low, which may be due to a lack of information or contradicting signals in this set of conserved loci. On the other hand, the mc4 dataset includes 68,499 loci with more than 80% missing data. Here *S. herbacea* occurs in sister position to the three major clades (I, II, III) (Figure 2). The two extremes in loci number reveal a bias of mainly conserved loci (mc58) on the one hand and the effects of too much missing data (mc4) on the other hand, which is in contrast to the findings of Eaton et al. (2017). We therefore suggest testing different thresholds to find the best settings for the specific group of interest.

RAD sequencing is the method that has made the most impact of phylogenetics so far (Andrews et al., 2016; McCormack, Hird, Zellmer, Carstens, & Brumfield, 2013). To analyze many small sequence fragments, covering the whole genome for many samples at low costs is a great advantage compared to traditional techniques. Our study confirms the utility of RAD sequencing data in resolving difficult phylogenetic problems where standard “fast‐evolving” markers have failed, joining several other examples (e.g., Jones et al., 2013; Wagner et al., 2013; Eaton & Ree, 2013; Wang et al., 2013; Escudero et al., 2014; Hipp et al., 2014; Ebel et al., 2015; Herrera & Shank, 2016; Mort et al., 2015; Vargas et al., 2017). We proved that RAD sequencing is a suitable tool to work on the species level as well as within and between subgenera in the genus *Salix*.

### 4.2 Relationships of subgenera *Chamaetia and Vetrix,* and the evolution of dwarf shrubs

Although the RAD sequencing analyses presented here are intended as a proof‐of‐concept for the ability of RAD sequencing to analyze infrageneric relationships on different levels of divergence, this preliminary analysis provides new biological insights into species relationships within subgenera *Chamaetia* and *Vetrix*. Our findings confirm the monophyly of the *Chamaetia/Vetrix* clade, but do not confirm the traditional treatment as two distinct subgenera, which was also shown by other molecular studies (Chen et al., 2010; Lauron‐Moreau et al., 2015; Wu et al., 2015). The traditional classification of the two subgenera was based on morphological characters like the number of stamens and nectar glands (Skvortsov, 1999). This subgeneric classification was accepted by some authors (Argus, 1973, 1997; Rechinger, 1964; Skvortsov, 1999), but the differences are minor and character states overlapping. For example, subgenus *Vetrix* is defined to have one nectar gland, whereas *Chamaetia* shows one or two nectar glands (Skvortsov, 1999). In his revision of the members of genus *Salix* in the New World, Argus (1973, 1997) accepted the split of the two subgenera, but added to the description of subgenus *Chamaetia* “the description does not fully separate *Salix* subg. *Chamaetia* from *Salix* subg. *Vetrix”* and the same sentence vice versa to the description of subgenus *Vetrix*. In the same line, Dorn (1976) only recognizes subg. *Vetrix* in an overview of American willows about 3 years later. Chou et al. (1984) as well as Fang, Zhao, and Skvortsov (1999) condoned the subgenera and used only the section level in an overview of Chinese *Salix* species. In a more recent study, Wu et al. (2015) confirmed the monophyly of the *Chamaetia/Vetrix* clade by traditional molecular data and inferred a crown group age of 23.76 Ma, but the subgenera were not reciprocally monophyletic. Based on these results, the authors suggest a merging of the two subgenera and therewith share the suggestions of Lauron‐Moreau et al. (2015), who conducted a similar approach on American willows. In a study investigating whole plastomes of Salicaceae, the three included species of *Chamaetia/Vetrix* also form a monophyletic group (Huang, Wang, Yang, Fan, & Chen, 2017). These results are in accordance with our findings focusing on European sections and species of both subgenera.

The five sections of subgenus *Chamaetia* were summarized because of their shared morphological and ecological similarities. This subgenus includes all species known as creeping willows or prostrate dwarf shrubs, showing a small growth habit less than 50 cm in height and occurring in high latitudinal or altitudinal ranges. This dwarf growth is due to the short vegetation period, low temperatures, and nutrient‐poor soils under alpine and arctic conditions (Körner, 2003). Dwarf growth is also adaptive for alpine woody plants, because the shoots remain under the snow cover during the winter and are protected from freezing damage. Moreover, the low stature of the plants creates a warmer temperature inside tissues (Körner, 2003). For these reasons, no taller shrubs—neither other willow species nor species of other plant families—do occur in arctic‐alpine tundra habitats. The most extreme form is shown by *S. herbacea,* a plant of snowbeds, which grows mostly with subterranean shoots and forms aboveground leafy shoots of only 1–2 cm height. *Salix serpillifolia*, a plant adapted to wind‐exposed alpine ridges, forms dense cushions as typical for such habitats. The decumbent *S. breviserrata* represents already a transition to a more erect growth up to 30 cm and is descending sometimes to the less harsh subalpine zone. These conspicuous and highly stable characters tempted authors to regard the dwarf shrubs as closely related. Despite the similarities of the included species, Skvortsov (1989, 1999) himself queried the monophyly of this subgenus and pointed out that the morphological distinctions between the two subgenera *Chamaetia* and *Vetrix* are minor. The author considered instead a convergent evolution of these characteristics as adaptations to the environmental and climatic conditions. However, the ancestral character state analyses (Figure 4) of our study indicates that dwarf shrubs evolved several times independently in *Salix* and thus is confirming the suggestions of Skvortsov (1989). The independent evolution of dwarf shrubs as an adaptation to the alpine zone was also observed in other groups, for example, in afro‐alpine *Alchemilla* species (Gehrke, Kandziora, & Pirie, 2016). However, the ability to form dwarf habits is present in several lineages of the *Chamaetia/Vetrix* clade and not an exclusive character of one subgenus only. This finding substantiates the combination of both subgenera to one clade. Nevertheless, Wu et al. (2015) suggested to maintain the nomenclatural separation into *Chamaetia* and *Vetrix* for practical reasons, although this is lacking any molecular evidence. We object this view and suggest to merge the two subgenera to *Chamaetia/Vetrix* clade, a nomenclature used in other studies as well. However, without a worldwide study covering all sections and species of *Chamaetia/Vetrix,* a new taxonomic classification of subgenera is nugatory. Our results suggest that infrageneric classifications on single morphological characters are probably unreliable because of homoplasy, which was also confirmed by findings of Wu et al. (2015) who revealed repeated reduction of number of stamens and a multiple origin of connate bud scales in subg. *Salix* s.l., both important characters used in traditional classification.

### 4.3 Relationships within the *Chamaetia/Vetrix* clade

Our data show a massive increase in phylogenetic resolution of the *Chamaetia/Vetrix* clade and give evidence to a split into three major clades. Every clade includes small dwarf shrubs to medium‐sized or big shrubs/trees. The species within the clades are all clearly monophyletic, independent of their geographic origin. The relationships between the species are well resolved and supported. In other studies based on one or few traditional plastid and/or nuclear markers, only low or no resolution could be observed and only one accession per species was included (Chen et al., 2010; Wu et al., 2015).

In the ML and BI analysis, the dwarf shrub species *S. reticulata* is sister to all remaining species (Figures 2 and 3). Similar results were presented in Liu, Wang, Wang, and Zhang (2016) based on two plastid markers (*mat*K and *rbc*L), but with low support. However, the sampling was much smaller and not all members of *Chamaetia/Vetrix* were sister to *S. reticulata* in their study. *S. reticulata* (Figure 1), a circumpolar arctic‐alpine species, is widely distributed and belongs together with four other arctic‐alpine species to section *Chamaetia* (Skvortsov, 1999). Their relationships, however, need to be tested.

The relationships within the *Chamaetia/Vetrix* clade do not reflect the taxonomic treatment, for example, the two species of section *Herbella* (syn. Sect. *Retusae*) included here*, S. herbacea* and *S. serpillifolia,* do not group together. This is in accordance with our character state evolution analysis (Figure 4) that revealed that morphological and ecological similar dwarf shrubs evolved several times independently. However, the species of the “*retusa* group” were suggested previously to be of different origin than the “*herbacea* group” by Rechinger (1964) and Ehrendorfer (1973), summarized in Hörandl (1992). Some authors put them in different sections (Chmelar & Meusel, 1986; Janchen, 1956). We did not include *S. retusa* in our phylogeny for it is polyploid, but our results confirm the treatment of diploid *S. herbacea* and *S. serpillifolia* as members of two distinct lineages.

### 4.4 Relationships on species level

All morphologically defined species were monophyletic in the ML and BI analyses, confirming the utility of the marker for species delimitation. Percy et al. (2014) included 546 individuals of 56 species in a study on two to seven plastid markers, but in contrast to our study, they failed to find species‐specific groups. In their study, some sections and species are not monophyletic, while other divergent species share the same haplotype. The three observed clades in our study contain species with adaptations to high altitudes as well as wide spread lowland species. So far we observed no apparent link between the species of each clade, neither morphologically, ecologically nor geographically. Wu et al. (2015) found similar results based on plastid data, where subclades could neither be explained by distribution pattern, morphological traits nor follow sectional classification. In our analysis, some species, such as *S. helvetica, S. foetida* or *S. breviserrata,* do have comparatively small distribution ranges in high‐mountain systems, others, such as *S. viminalis* or *S. purpurea,* are widely distributed lowland species. The distribution ranges of the species are partly overlapping, but in many cases the ecological niche is different (Schiechtl, 1992; Skvortsov, 1999). However, hybridization events are well‐known between *Salix* species, especially in (secondary) contact zones, for example, between *S. purpurea* and *S. helvetica* (Gramlich & Hörandl, 2016; Gramlich, Sagmeister, Dullinger, Hadacek, & Hörandl, 2016). Strikingly, these two species belong to different subclades, indicating the potential of hybridization across the whole clade. To avoid a bias due to hybrids, we included only purebred species with clear morphological characters in our dataset, and our results of well‐supported monophyletic species groups confirm our conservative species identification. Nevertheless, recent analyses showed that reticulate evolution seems to play an important role in *Salix* evolution, for example, hybridization/introgression (Gramlich, 2017; Percy et al., 2014). Genetic introgression is not necessarily visible in morphological characters. While showing a distinctive morphology, individuals may share genetic portions with their putative hybrid partners after backcrossing events. Given the huge number of RAD loci, it might be possible that introgressed loci may have played a role in our phylogenetic reconstructions leading to genetic clustering of frequently hybridizing species (e.g., *S. foetida* and *S. helvetica*). However, this alone cannot explain the overall pattern of the phylogeny, for other species also frequently form natural hybrids without grouping in the same clade. We therefore rather suggest ancient hybridization and introgression pattern as a reason of the configuration of clades in our phylogenies.

Despite the monophyletic species‐specific clades in the ML and BI phylogenies, the genetic structure analysis showed that some species share the same genetic cluster (Figure 5). This very low level of genetic divergence may be a result of reticulate evolution as a consequence of recent or ancient gene flow as was found in other studies on willow evolution (Percy et al., 2014; Wu et al., 2015). To guarantee the congruency of datasets in this study, only loci shared by at least 20 individuals (mc20) were taken into account for the structure analyses and only unlinked SNPs, that is, one SNP per locus, were used. This decreases the putative intraspecific variation. Loci present (and putatively variable) only within a species were discarded during the pipeline. Hence, the structure analysis including all species mostly reflects more ancient polymorphisms and does not yield species‐specific clusters. An influence of recent introgression is unlikely as some clusters contain species which do not share the same ecological niche (Schiechtl, 1992), for example, the lowland species *S. viminalis* never occurs at the same locality as the alpine species *S. helvetica* and *S. foetida*. Additionally, no admixture is observed as would be expected with recent hybridization/introgression events. However, this suggestion needs further testing, because frequent hybridization is known among willows, which are mainly insect pollinated and have small wind or water dispersed seeds leading to effective pollen transfer and seed dispersal over long distances (Argus, 1997; Percy et al., 2014).

The morphologically well‐defined and ecologically distinct species are in contrast to the genomic similarity of each cluster and clade in the phylogenies, respectively. When using more loci and a clade‐specific pipeline (using loci shared by 2 individuals), each species shows a unique genetic cluster, but the genetic structure within the four accessions of each species is still low (data not shown). However, intraspecific variation patterns were not the focus of this study. Interestingly, *S. herbacea* shows as only diploid lineage not a single genetic cluster but some genetic admixture in the structure analysis, although the pattern within this species is uniform (Figure 5). The identical genetic structure of samples from Norway and the Alps confirms the findings of Alsos, Alm, Normand, and Brochmann (2009) that proposed a continuous distribution and frequent gene flow of *S. herbacea* between Northern Europe and the Alps during the last glacial maximum. Although the distribution is now disjunct, the European populations share the same ancestral history, which is also reflected in our molecular data. The observed admixture likely reflects ancient hybrid evolution or introgression. Genetic admixture in the triploid *S. bicolor* may indicate an allopolyploid origin.

## 5 CONCLUSION

We presented the first well‐resolved phylogeny of European members of *Salix Chamaetia/Vetrix* clade based on RAD sequencing and a comprehensive sample set covering 13 sections. The method has proved to be suitable to implement phylogenetic relationships from the subgenus to species level in this taxonomically difficult group of shrub willows. We could infer the relationships between the clearly delimited species, but the results revealed that the classical infrageneric taxonomic treatment is not supported by molecular data, neither subgenera nor sections were monophyletic. We therefore suggest to merge the two subgenera to “*Chamaetia/Vetrix* clade” until a more comprehensive phylogeny and a new taxonomic revision of this group is available. We further revealed an independent evolution of dwarf shrubs within *Chamaetia/Vetrix* for at least four times. Although our sampling is not complete, we can present a first phylogenetic overview as a framework for further studies on sister relationships, hybridization events, and other evolutionary studies. The next step would be to include more species and sections of the *Chamaetia/Vetrix* clade, also including the polyploid species, to reveal more detailed information on the evolution of European shrub willows.

## AUTHOR CONTRIBUTIONS

E. H. and N. D. W. designed the research, S.G. did sampling and laboratory work, N. D. W. performed the research and analyzed the data, N. D. W. wrote the manuscript with assistance of S. G. and E. H.

## DATA ACCESSIBILITY

All demultiplexed read data were submitted to the National Center for Biotechnology Information SequenceReadArchive: BioProject ID PRJNA433286.
