# Supplementary material for: RAD sequencing resolved phylogenetic relationships in European shrub willows (Salix L. subg. Chamaetia and subg. Vetrix) and revealed multiple evolution of dwarf shrubs
Source: Ecol Evol. 2018 Jul 22;8(16):8243–55. doi: 10.1002/ece3.4360 (PMC6145212; doi:10.1002/ece3.4360)
Supplement: Supplementary file 1 [file ECE3-8-8243-s001.pdf]

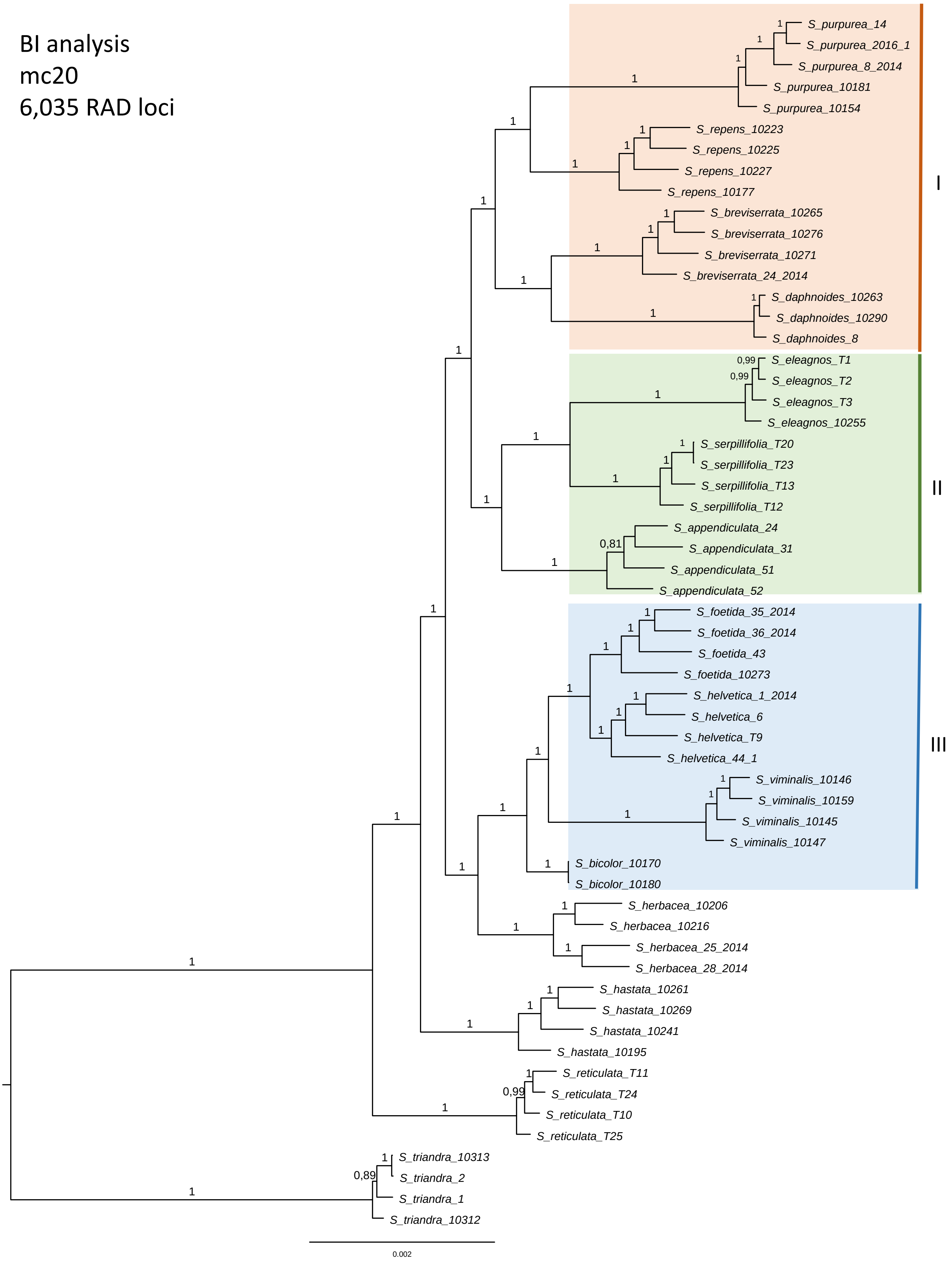

Fig. S1 Phylogenetic trees inferred from the Bayesian inference analysis of the *Salix Chamaetia/Vetrix* clade of the mc20, mc35 (b), mc50 (c), and mc58 (d) RAD sequencing data sets. *S. triandra* was used as outgroup. Posterior probability (PP) values are given above branches. The three main clades are indicated by coloured boxes.

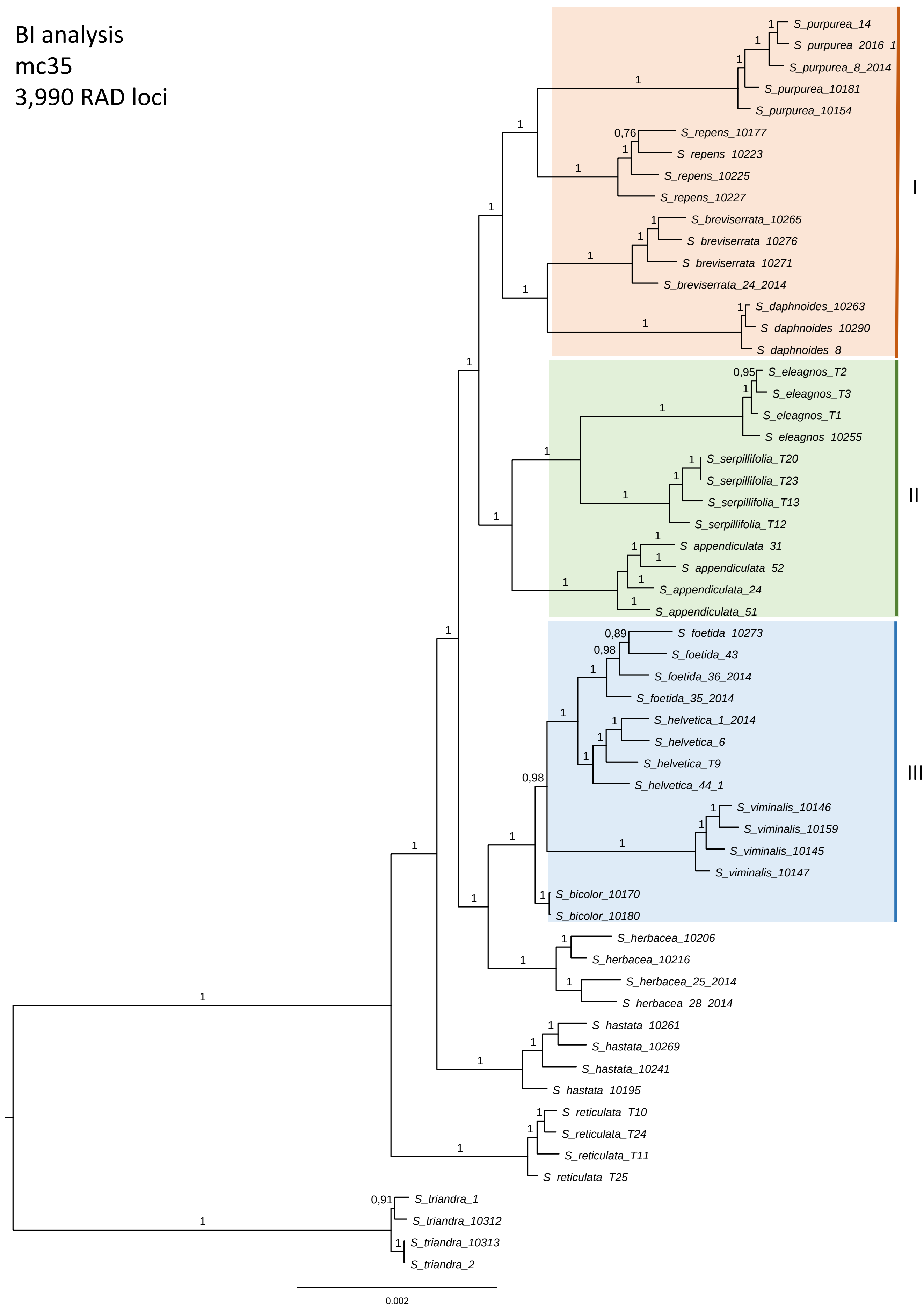

Fig. S1(b) Bayesian inference analysis of the mc35 RAD data set

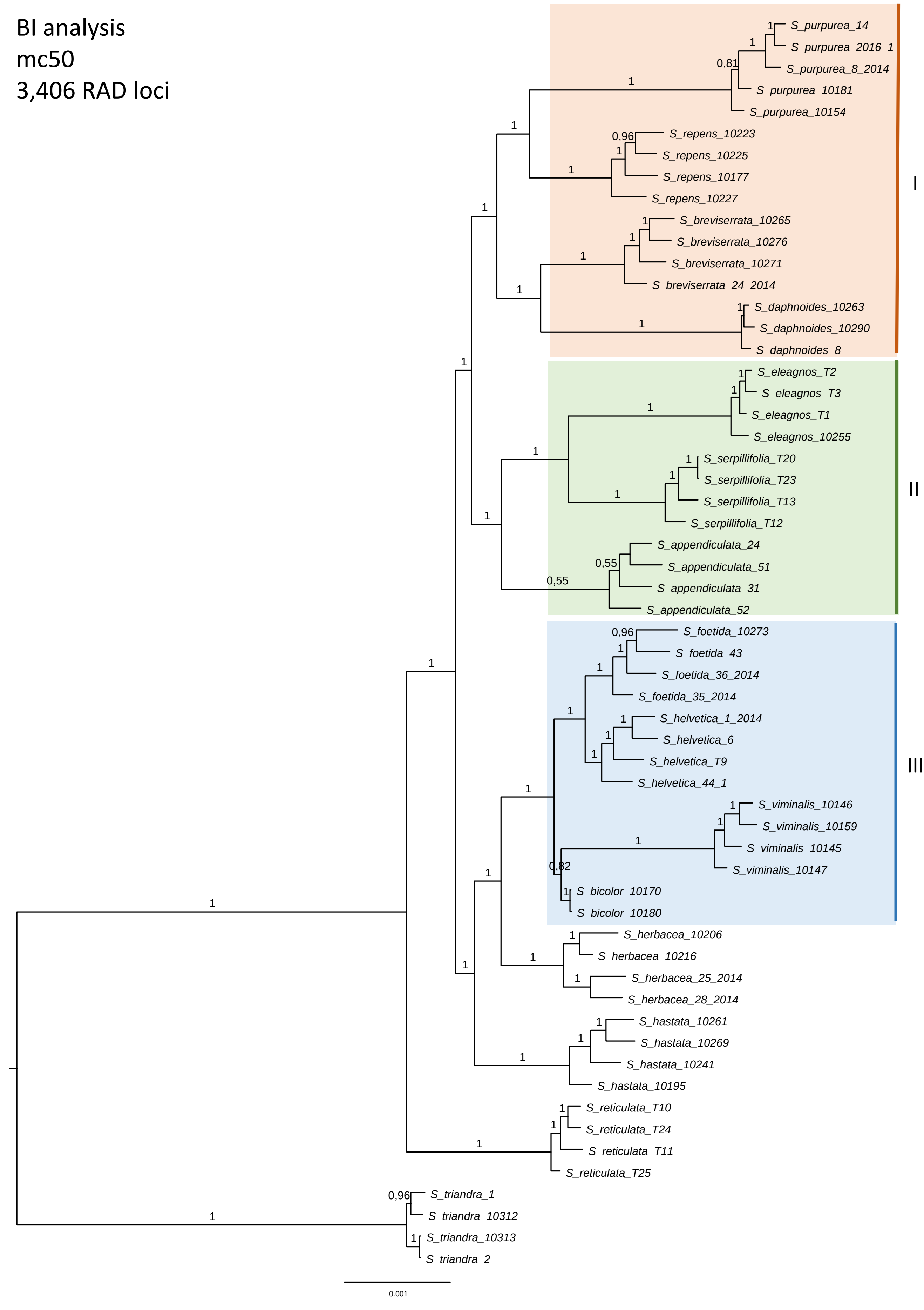

Fig. S1(c) Bayesian inference analysis of the mc50 RAD data set

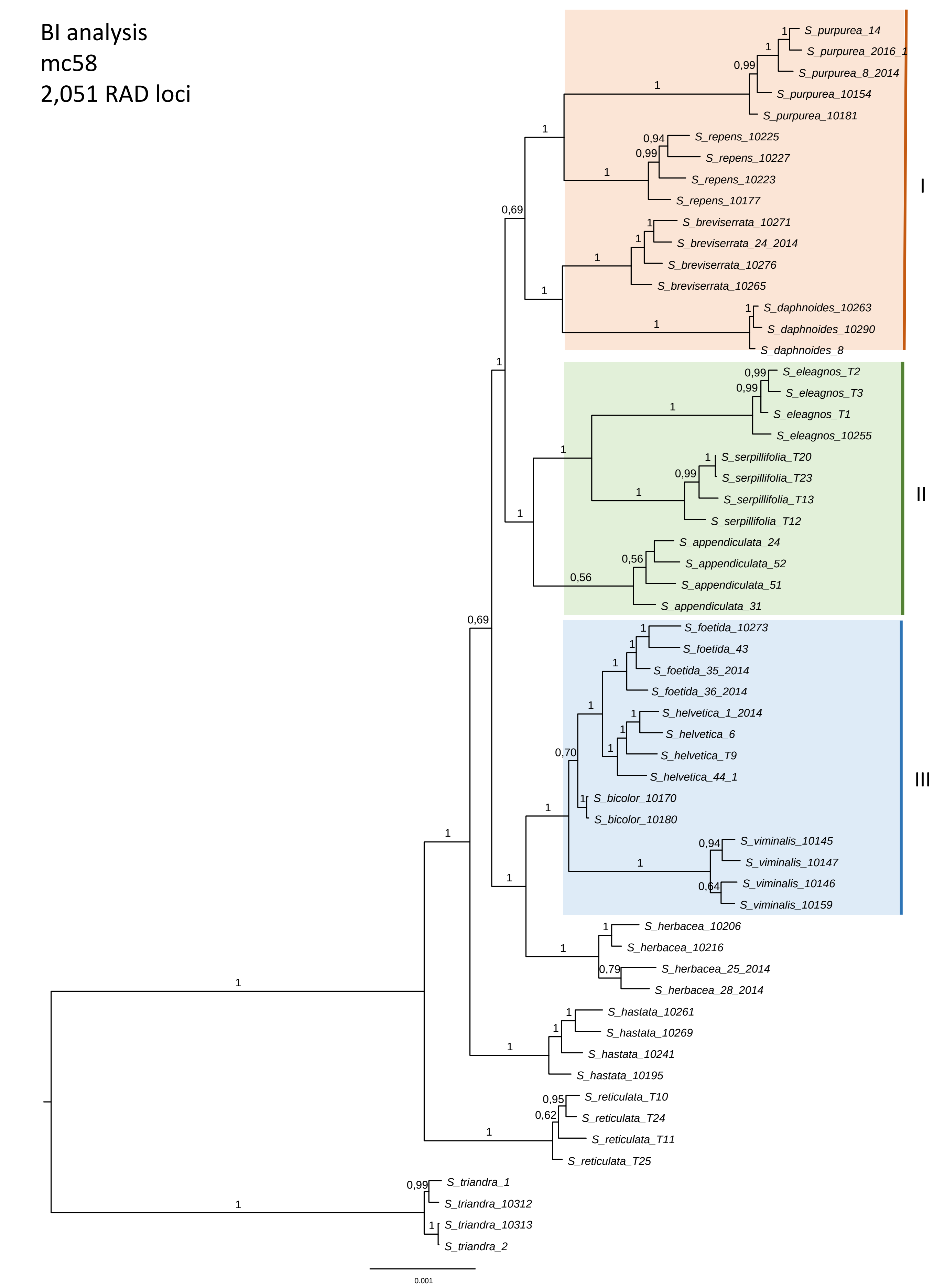

Fig. S1(d) Bayesian inference analysis of the mc58 RAD data set
